# Circular RNA circEGFR regulates tumor progression via the miR-106a-5p/DDX5 axis in colorectal cancer

**DOI:** 10.1590/1414-431X2020e10940

**Published:** 2021-07-23

**Authors:** Ping Fu, Liangqing Lin, Hui Zhou, Sijun Zhao, Zhigang Jie

**Affiliations:** 1 Department of General Surgery, Jiangxi Provincial People's Hospital Affiliated to Nanchang University, Nanchang, Jiangxi, China; 2 Department of General Surgery, The First Affiliated Hospital of Nanchang University, Nanchang, Jiangxi, China

**Keywords:** circEGFR, Colorectal cancer, Progression, DDX5, miR-106a-5p

## Abstract

Recently, an increasing number of studies have reported that dysregulation of circular RNA (circRNA) expression plays critical roles in the progression of several cancers, including colorectal cancer (CRC). However, the detailed molecular mechanisms of circRNAs involvement in CRC remain largely unknown. Here, we confirmed that the level of circEGFR was significantly increased in CRC tissues compared to matched adjacent non-tumor tissues, and a high level of circEGFR was correlated with poor clinicopathological characteristics and poor prognosis in patients with CRC. Moreover, increased circEGFR expression promoted CRC cell proliferation, migration, and invasion *in vitro*. Mechanistically, circEGFR acted as a ceRNA for miR-106a-5p to relieve the repressive effect of miR-106a-5p on DDX5 mRNA. Moreover, circEGFR enhanced DDX5 expression, thereby upregulating p-AKT levels. Together, these findings showed that circEGFR promoted CRC cell proliferation, migration, and invasion through the miR-106a-5p/DDX5/AKT axis, and may serve as a promising diagnostic marker and therapeutic target for CRC patients.

## Introduction

Colorectal cancer (CRC) is the third most lethal malignancy worldwide and is responsible for the second most frequent cause of cancer deaths each year (1,2). High frequencies of relapse and metastasis contribute to the poor prognosis of CRC patients. Despite advances in surgery, chemotherapy, targeted therapy, and immunotherapy for CRC, the 5-year survival rate for patients with relapse and metastasis is still poor (3). Therefore, exploring the biological molecular mechanisms underlying CRC relapse and metastasis is extremely urgent, as it may provide promising targets for diagnosis and therapy.

Circular RNAs (circRNAs) are a class of RNAs with or without protein coding ability that are widely dysregulated and exist in different cancers (4,5). Their structure contains a covalently closed loop without a poly-A tail at the 3' end and without a cap structure at the 5' end. Due to this specific structure, circRNA can avoid degradation by nucleic acid exonucleases and has a much longer half-life than linear mRNA (48 h *vs* 10 h). In addition, it is also highly conserved, and thus there is a relationship between the dysregulation of its expression and the development of various diseases (including various cancers) (4-6). Recently, an increasing number of studies have also identified many circRNAs that contribute to tumorigenesis and can directly regulate transcription and translation processes by interacting with mRNAs and miRNAs (7,8). Among them, circRNAs were found to affect tumorigenesis by sponging miRNAs to regulate the function of target genes. However, their biological functions are poorly understood. To date, several studies have reported the biological role of circRNAs in CRC. For example, Han et al. (9) reported that circLONP2 enhances invasion and metastasis of CRC cells. Pei et al. (10) confirmed that circ_0000218, as a sponge for miR-139-3p, is able to inhibit CRC progression. These studies make circRNAs promising prognostic biomarkers and therapeutic targets in the treatment of CRC.

DDX5 is a prototypical member of the DEAD box family of RNA helicases and plays important roles in multiple biological processes, including cell proliferation, early organ development, and maturation (11,12). Shin et al. have reported that DDX5 was overexpressed in CRC, and it was associated with the progression of cancers (13). Furthermore, several studies have shown that miRNAs can regulate the function of DDX5, such as the interaction of DDX5 with miR-28-5p in prostate cancer (14), or miR-431 regulation of the function of DDX5 in lung cancer (15).

A previous study has confirmed that circEGFR may play an important role in ovarian granulosa cells by modulating Fyn via competitive binding with miR-125a-3p (16). However, the mechanism of circEGFR in CRC has not been studied. Here, we confirmed that circEGFR expression was frequently increased in CRC tissues compared with paired adjacent non-tumor tissues, and patients with higher circEGFR expression had a poorer prognosis. Mechanistically, circEGFR has biological functions in CRC by sponging miR-106a-5p to upregulate DDX5 expression and promote CRC cell proliferation and invasion.

## Material and Methods

### Patients and tissues

A total of 90 paired CRC tissues and adjacent non-tumor tissues were obtained from patients with CRC at the Jiangxi Provincial People's Hospital. None of the patients had received therapy before surgery. This study was approved by the Institutional Ethical Committee of the Jiangxi Provincial People's Hospital. Written informed consent was obtained from every patient. The information of CRC patients is shown in Supplementary Table S1.

### Cell culture

The human CRC cell lines HCT-116, HT-29, SW480, and LOVO were purchased from the Institute of Biochemistry and Cell Biology of the Chinese Academy of Sciences (China). All cells were cultured in Dulbecco's modified Eagle's medium supplemented with 10% of fetal bovine serum (FBS), 100 IU/mL penicillin, and 100 μg/mL streptomycin at 37°C in a humidified incubator with 5% CO_2_.

### Transfection

The transfection process was performed with Lipofectamine 2000 (Invitrogen, USA) following the manufacturer's instructions. The miRNA mimics were chemically synthesized by GenePharma (China), double-stranded RNAS that mimic mature endogenous miRNAS after transfection into cells. Overexpression and short hairpin RNA adenovirus were purchased from Genomeditech (China).

### qRT-PCR and western blotting

An ABI PRISM 7900 Sequence Detection System (Applied Biosystems, USA) was used for relative gene amplification and detection. The quantitative real-time polymerase chain reaction (qRT-PCR) process was performed as described in a previous study (4). GAPDH and U6 served as internal controls. The primers were designed as follows: circEGFR: F (forward) 5′-CGGGACATAGTCAGCAGTGA-3′, R (reverse) 5′-ACATCCTCTGGAGGCTGAGA-3′; miR-106a-5p: F 5′-GATGCTCAAAAAGTGCTTACAGTGCA-3′, R 5′-TATGGTTGTTCTGCTCTCTGTCTC-3′; U6: F 5′-GCTTCGGCAGCACATATACTAAAAT-3′, R 5′-CGCTTCACGAATT TGCGTGTCAT-3′; DDX5: F 5′-GCCGGGACCGAGGGTTTGGT-3′, R 5′-CTTGTGCTGTGCGCCTAGCCA-3′; GAPDH: F 5′- CAATGACCCCTTCATTGACC-3′, R 5′-GACAAGCTTCCCGTTCTCAG-3′ (Sangon, China). For western blotting analysis, RIPA buffer (Beyotime, China) was used to extract the total cell protein, and then, a BCA kit (Beyotime) was used to detect the protein concentration. The proteins were separated by sodium dodecyl sulfate-polyacrylamide gel electrophoresis (SDS-PAGE) and transferred onto the nitrocellulose membranes. Then, the membranes were incubated overnight at 4°C with AKT (#2920), p-AKT (#4051), DDX5 (#9877) antibodies (1:1000, Cell Signaling Technology, USA), and β-actin antibodies (sc-8432) (1:1000, Santa Cruz, USA) after blocking with 5% skim milk. The membranes were then incubated with the HRP-conjugated secondary antibody (A0208 and A0216) (1:5000, Beyotime) for 1 h. Finally, the expression of immune complexes was detected with enhanced chemiluminescence reagents (Pierce, USA) (17).

### Cell proliferation assay and clone formation assay

A Cell Counting Kit-8 (CCK-8, Yeasen, China) was used to assess the proliferative ability of CRC cells. Then, 100 μL culture media containing 1000 transfected or untransfected cells was seeded onto each well of a 96-well plate, and CCK-8 reagent (10 μL) was added to each well for the next 24, 48, 72, 96, and 120 h. The absorbance was assessed at 450 nm. For the clone formation assay, a thousand CRC cells and transfected cells were inoculated onto cell culture plates. Then, the cells were washed with PBS and fixed with 4% paraformaldehyde after culturing for 14 days. After that, the cells were stained with crystal violet and the number of clones was counted. All the experiments were performed three times.

### Transwell assay

Transwell plates (24-well) were used for invasion experiments. CRC cells and transfected cells (10,000) were seeded onto the upper chamber containing 200 μL serum-free medium. Then, 500 μL of medium containing 20% FBS was added to the lower chamber. Cells were fixed with 4% paraformaldehyde and stained with crystal violet after culturing for 48 h. A cotton swab was used to wipe the upper cell layer and the number of cells was counted under a microscope (Olympus (Beijing) Sales Service Co., Ltd., China).

### Luciferase reporter assays

Wild-type and mutated circEGFR miRNA binding sequences were inserted into the KpnI and SacI sites of a pGL3 promoter vector for luciferase reporter assays (Genomeditech). The vectors were co-transfected into CRC cells using Lipofectamine 2000 (Invitrogen) according to the manufacturer's instructions. The cells were harvested at 48 h and the luciferase activity was assessed using a dual-luciferase reporter assay (Promega, USA). The wild-type circEGFR miRNA binding sequence is 5′-GCUCACGCAGUUGGGCACUUUU-3′ and the mutated circEGFR miRNA binding sequence is 5′-GCUCACCGUGAAGGCGUGAAAU-3′.

### Statistical analysis

IBM SPSS software (21.0; SPSS, Inc, USA) was used for the Student's *t*-tests, the correlation analysis, the chi-squared test, Kaplan-Meier analyses, and the log-rank test. GraphPad Prism 7.0 (USA) was used to produce the diagrams and charts. P<0.05 was considered statistically significant.

## Results

### circEGFR expression in CRC tissues and correlation with prognosis

First, we analyzed the EGFR-derived circRNA expression in 4 CRC tissues and paired adjacent non-tumor tissues using RT-qPCR. The results showed that hsa_circ_0080220 was upregulated in all the 4 CRC tissues compared with paired adjacent non-tumor tissues. Hence, we have termed hsa_circ_0080220 as “circEGFR”.

**Figure 1:**
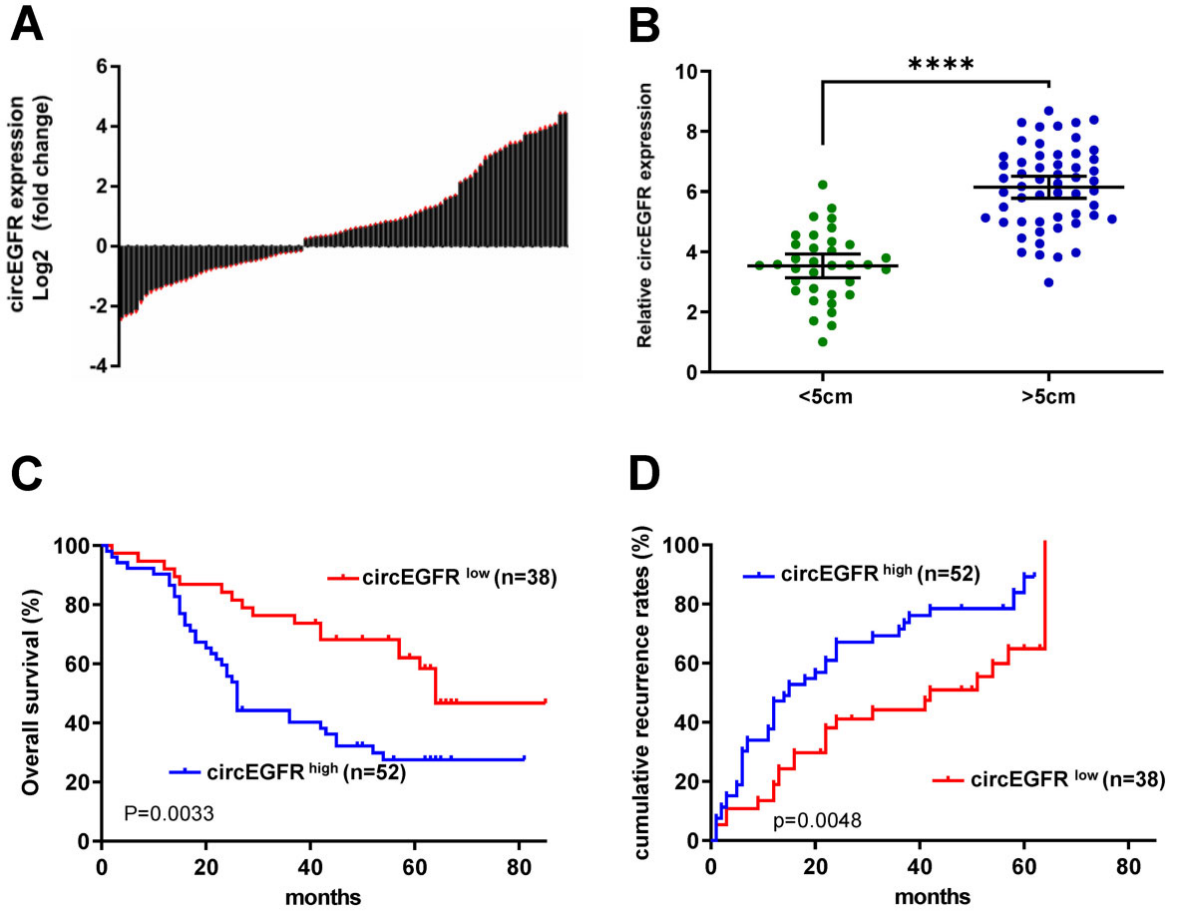
circEGFR is overexpressed in colorectal cancer (CRC) tissues and is correlated with poor prognosis. **A**, circEGFR expression in 90 paired CRC tissues and their adjacent normal tissues. **B**, circEGFR expression is upregulated in larger tumors. Data are reported as means±SD. ****P<0.0001 (unpaired *t*-test). **C** and **D**, The higher circEGFR expression is correlated with poor prognosis in CRC patients (overall survival: P=0.0033; recurrence rate: P=0.0048, Kaplan-Meier).

circEGFR expression was significantly increased in CRC tissues compared to adjacent non-tumor tissues (52/90) (Figure 1A). Clinically, the circEGFR expression was significantly higher in larger tumors (>5 cm) than in smaller tumors (≤5 cm) (Figure 1B). Then, we divided the 90 patients into circEGFR^high^ (not all large tumors) and circEGFR^low^ groups (tumors of different sizes). The Kaplan-Meier analysis and log-rank test results showed that increased circEGFR expression was associated with poor prognosis in CRC patients in terms of overall survival and cumulative recurrence after surgery (Figure 1C and D).

### circEGFR expression and CRC cell proliferation, migration, and invasion

**Figure 2:**
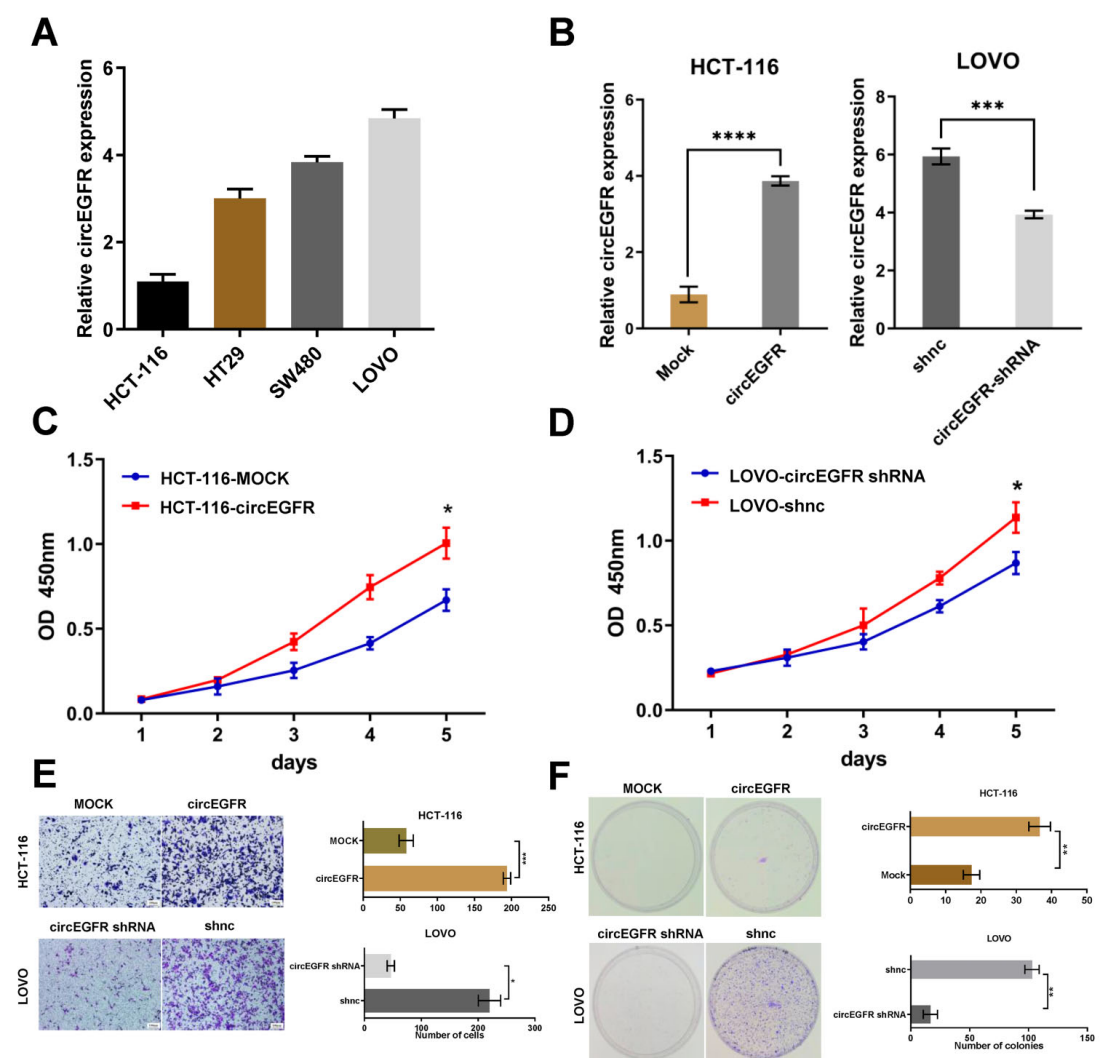
Increased circEGFR expression promoted proliferation, migration, and invasion of colorectal cancer (CRC) cells *in vitro*. **A**, The expression level of circEGFR in CRC cell lines. **B**, The circEGFR expression level in HCT-116 and LOVO cells after transfection with circEGFR overexpression and shRNA lentivirus. **C** and **D**, The cell viability of HCT-116-circEGFR and LOVO-circEGFR shRNA was assessed by CCK-8 assay. **E**, Transwell assays were performed to assess the invasion ability of HCT-116-circEGFR and LOVO-circEGFR shRNA cells. Scale bar: 100 μm. **F**, Colony formation assays were performed to assess the proliferative ability of CRC cells. Data are reported as means±SD. *P<0.05; **P<0.01; ***P<0.001; ****P<0.0001 (*t*-test and ANOVA).

Loss-of-function and gain-of-function assays were carried out to explore whether circEGFR could affect the biological function of CRC cells. Since HCT-116 cells expressed the lowest level of circEGFR and LOVO cells expressed the highest level of circEGFR, these cell lines were chosen for further study (Figure 2A). As shown in Figure 2B, transfection with circEGFR overexpression lentivirus markedly increased the circEGFR levels in HCT-116 cells, and transfection with circEGFR shRNA lentivirus markedly decreased the circEGFR levels in LOVO cells. Compared with the mock-transfected cells, HCT-116-circEGFR cells exhibited significantly increased viability according to CCK-8 assays (Figure 2C). Conversely, compared with the negative control (NC) group, LOVO-sh-circEGFR cells exhibited significantly decreased viability (Figure 2D). Furthermore, Matrigel transwell analysis was performed to determine whether circEGFR could affect the invasion ability of CRC cells. The invasion ability of HCT-116 cells was increased in the circEGFR-overexpressing group, and the invasion ability of LOVO cells was decreased in the circEGFR-knockdown group (Figure 2E). Moreover, colony formation assays showed that the proliferative ability of HCT-116 cells was increased in the circEGFR-overexpressing group and that of LOVO cells was decreased in the circEGFR-knockdown group (Figure 2F).

### circEGFR functioned as a ceRNA to regulate DDX5 expression in CRC

**Figure 3:**
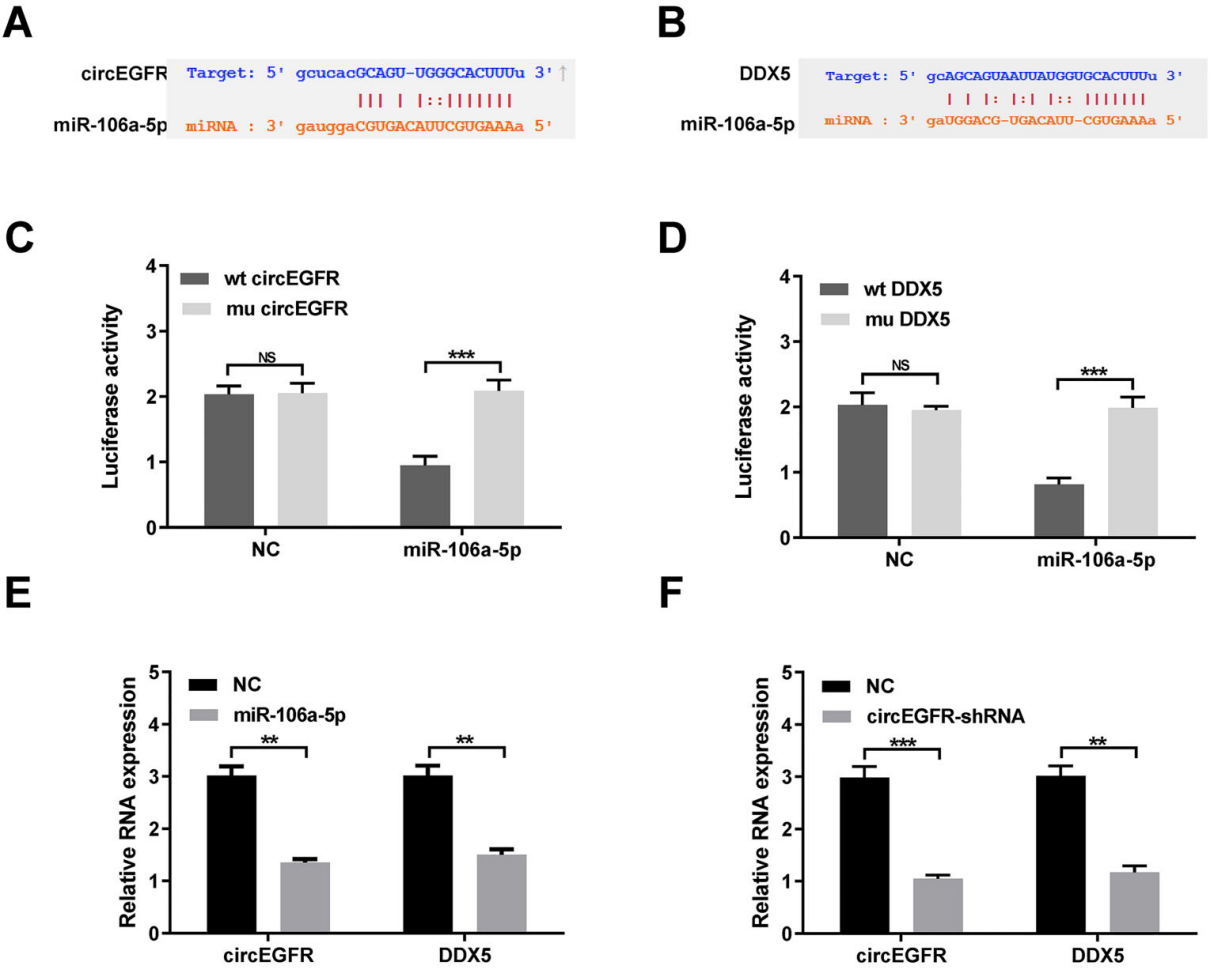
circEGFR functions as a ceRNA to regulate DDX5 expression in colorectal cancer (CRC). **A** and **B**, The potential miRNA and its target gene sequences were explored by starBase 3.0 software. **C** and **D**, The relative luciferase activity was detected in CRC cells co-transfected with wild-type (wt) or mutated-type (mu) circEGFR or DDX5 3′-UTR and miR-106a-5p mimic. **E**, DDX5 expression was significantly increased after overexpression of circEGFR. **F**, DDX5 expression was significantly reduced after the knockdown of circEGFR. Data are reported as means±SD. **P<0.01; ***P<0.001 (*t*-test). NS: not significant; NC: negative control.

Recently, several studies have shown that circRNAs can serve as competing endogenous RNA (ceRNA) sponges of miRNAs in many malignant tumors including CRC (4,6,18,19). Here, starBase 3.0 (https//bio.tools) was used to explore potential target miRNAs of circEGFR, and the results revealed miR-106a-5p as a predicted target miRNA (Figure 3A). A previous study reported that forced DDX5 expression promotes carcinogenesis and progression in several tumors (20,21). Therefore, miR-106a-5p attracted our attention, as it might directly target DDX5 (Figure 3B). Next, we investigated whether circEGFR and DDX5 mRNA 3′UTR could bind to miR-106a-5p. To further verify these predicted interactions, wild-type (wt) and mutant (mu) circEGFR and DDX5 mRNA 3′UTR luciferase reporter plasmids (pLG3 vector) were used. Luciferase reporter plasmids containing a wild type or mutant circEGFR and DDX5 mRNA 3′UTR were co-transfected with miR-106a-5p mimics or NC into HCT-116 cells. The relative luciferase activity was significantly lower in HCT-116 cells co-transfected with the wild type circEGFR or DDX5 mRNA 3′UTR luciferase reporter and miR-106a-5p mimic than in the control groups (Figure 3C and D). Importantly, DDX5 mRNA expression was significantly increased after overexpression of circEGFR (Figure 3E). In contrast, DDX5 mRNA expression in the circEGFR knockdown group was significantly reduced compared with that in the negative control cells (Figure 3F).

### circEGFR regulated the expression of the DDX5 and the DDX5-mediated signaling pathway in CRC

**Figure 4:**
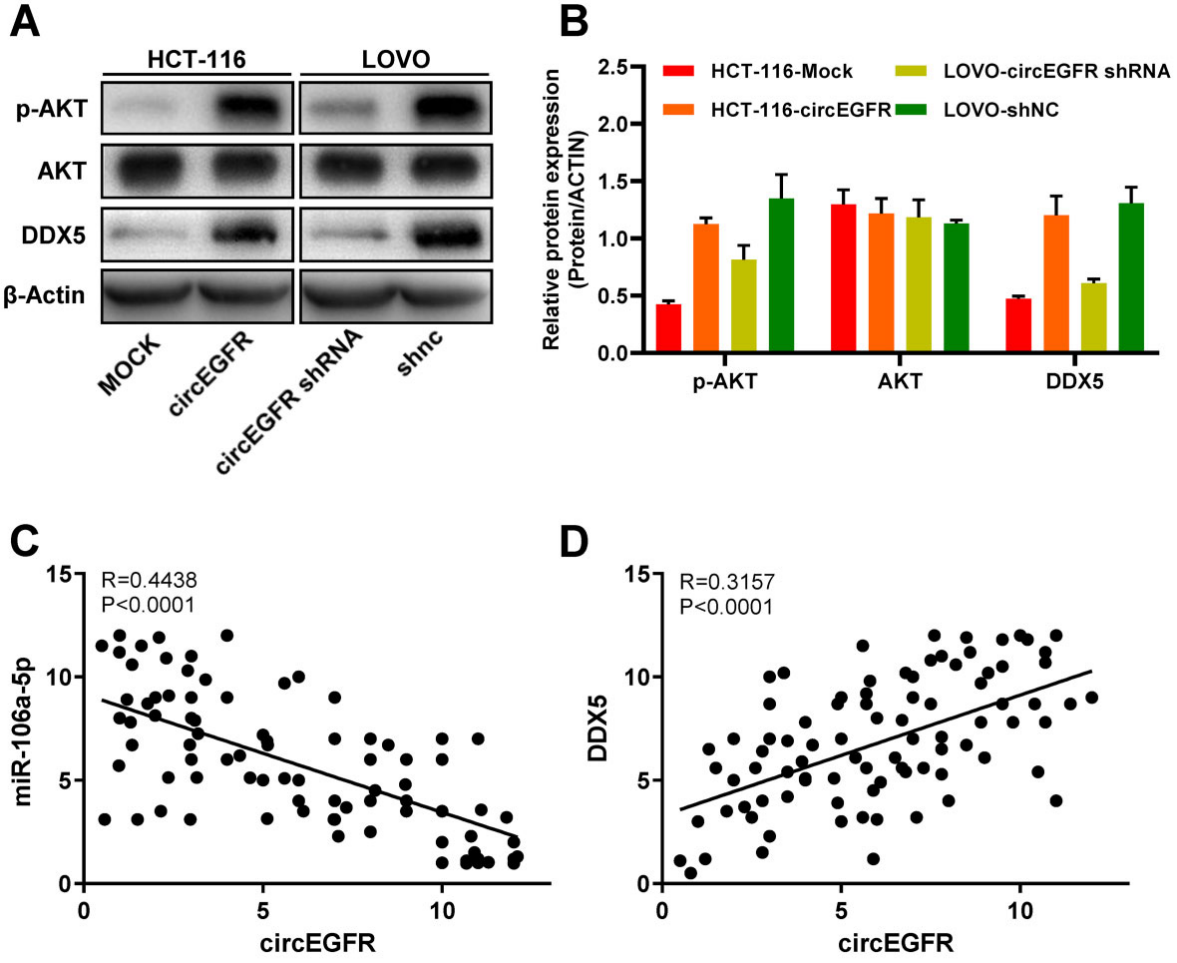
circEGFR regulates the expression of the DDX5 and the DDX5-mediated signaling pathway in colorectal cancer (CRC). **A** and **B**, circEGFR overexpression upregulated the level of p-AKT while circEGFR knockdown reduced the p-AKT expression; actin served as internal control. Data are reported as means±SD. **C** and **D**, A positive correlation was observed between circEGFR and DDX5 while a negative correlation was observed between circEGFR and miR-106a-5p.

The AKT pathway is activated abnormally in several types of cancers with increased expression of DDX-5 and is closely related to cancer progression (16,22,23). In this study, we speculated that circEGFR may enhance CRC cell proliferation and invasion via the AKT signaling pathway. The downregulation of the circEGFR expression markedly reduced the expression of phosphorylated-AKT (p‐AKT), whereas increased circEGFR expression upregulated the level of p‐AKT (Figure 4A and B). However, the total AKT expression showed no significant change (Figure 4A and B). To further verify whether circEGFR upregulates the DDX5 expression by sponging miR‐106a‐5p, we measured the expression of miR‐106a‐5p and DDX5 in tissues from the 70 CRC tissue samples. The results showed that there was a negative correlation between circEGFR and miR‐106a‐5p in the CRC tissues. Moreover, positive correlations between circEGFR and DDX5 were observed in the CRC tissues (Figure 4C and D).

### DDX5 knockdown affected the circEGFR-induced CRC progression

**Figure 5:**
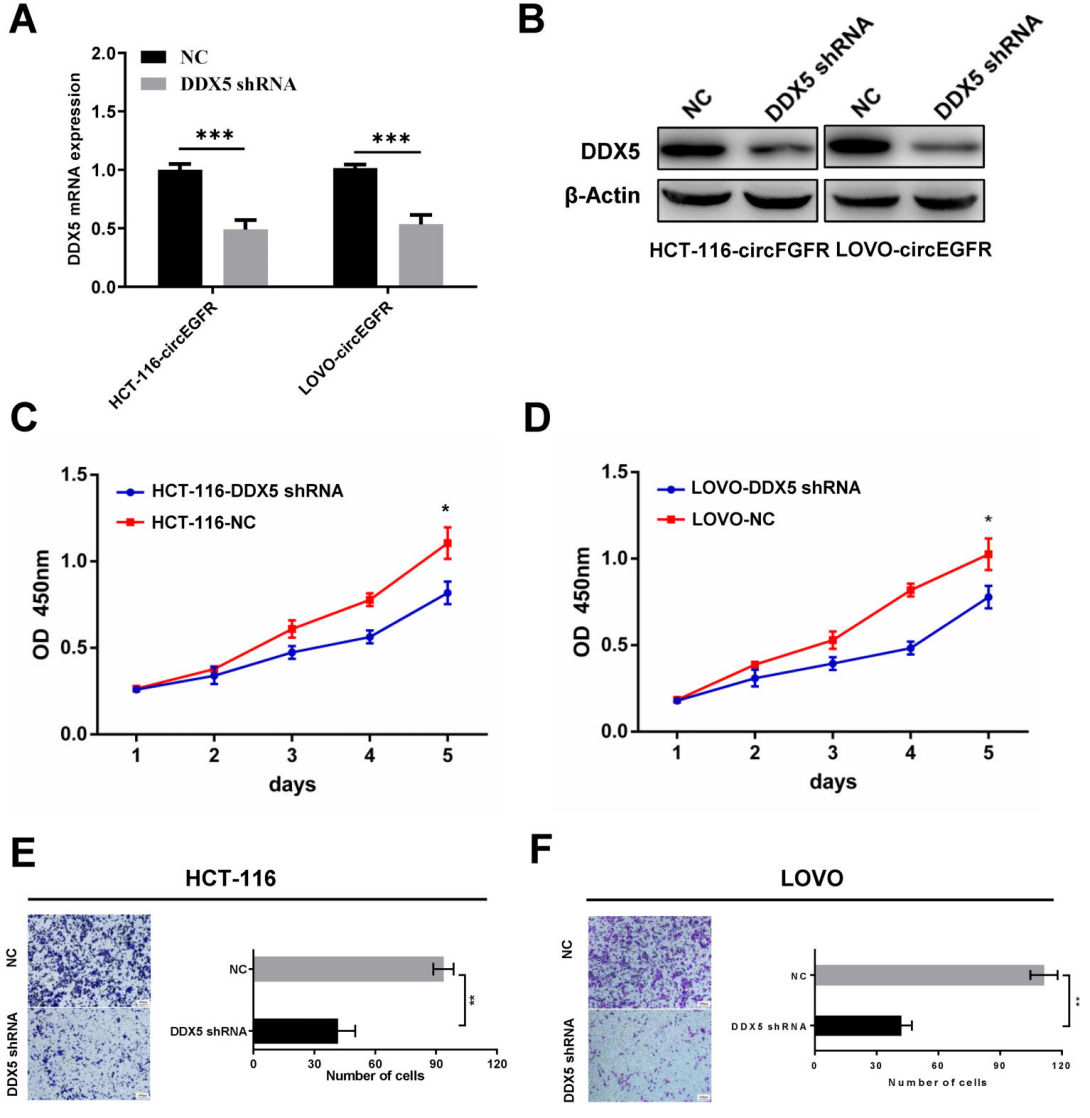
DDX5 knockdown affected the circEGFR-induced colorectal cancer (CRC) progression. **A**, qRT-PCR was performed to verify the knockdown efficacy of DDX5. **B**, Western blot was performed to validate the knockdown efficacy of DDX5, and actin served as the internal control. **C** and **D**, The proliferative abilities of HCT-116 and LOVO cells were determined using CCK-8 assays, and downregulated DDX5 suppressed the proliferation of HCT-116 and LOVO cells. **E** and **F**, The invasion abilities of HCT-116 and LOVO cells was assessed by transwell assays, and downregulated DDX5 suppressed the invasion of HCT-116 and LOVO cells (scale bar 100 μm). Data are reported as means±SD. *P<0.05, **P<0.01, ***P<0.001 (*t*-test). NC: negative control.

**Figure 6:**
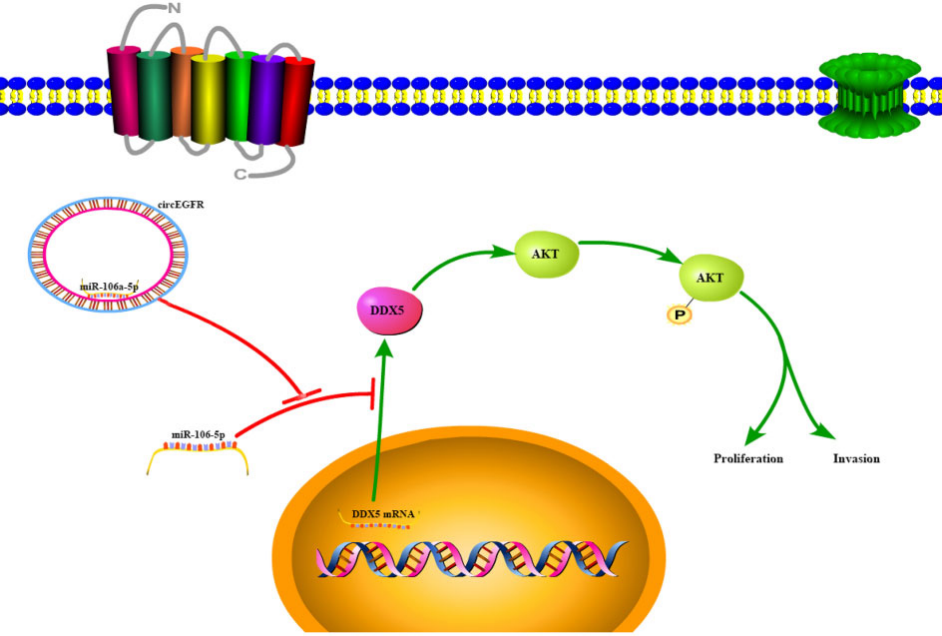
Working model and mechanism diagram of colorectal cancer cell proliferation and invasion.

circEGFR promoted the DDX5 expression and activated AKT signaling in CRC cells, therefore, we investigated whether the oncogenic functions of circEGFR could be inhibited by DDX5 knockdown. We first transfected a DDX5 shRNA plasmid into HCT-116-circEGFR and LOVO-circEGFR cells. The shRNA knockdown efficacy was validated by qRT-PCR and western blot analysis (Figure 5A and B). Reduced DDX5 inhibited the circEGFR overexpression-mediated AKT phosphorylation and cell proliferation and invasion *in vitro* (Figure 5C-F) (22,24). These results indicated that circEGFR might regulate the AKT pathway by upregulating the DDX5 expression (Figure 6).

## Discussion

CRC patients have continued to suffer high recurrence rates and mortality (25). The pathogenic mechanism underlying CRC is also elusive. Although CRC is a serious health problem, there are few interventions available for this disease. Therefore, our goal was to further determine the pathogenic genes promoting the development of CRC.

Recent studies point to the carcinogenesis of circRNA in many types of cancer, such as circRNA_100290 in oral cancer (26), circHIPK3 in bladder cancer (27), and circRNA CCDC66 in colon cancer (28), etc. A number of genes including circRNAs have been found to participate in ceRNA networks (29,30). A previous study has established the circRNA-miRNA-mRNA regulatory framework, which is of great importance to further substantiate the function of circRNAs and their target genes (31). circEGFR as a conserved RNA is more stable than linear RNA in certain challenging environments (such as in RNase R digestion experiment), and it is also overexpressed in ovarian cell lines (16). In addition, the upregulation of circEGFR can affect the proliferation of granulosa cells. Because of the functions of circRNA, we hypothesized that circEGFR may be related to many diseases. Our study confirmed that the circEGFR expression in CRC tissues was significantly higher than that in the adjacent normal tissues. The expression of circEGFR was highly associated with CRC patient prognosis. High circEGFR expression was correlated with poor prognosis and a high recurrence rate. *In vitro*, we demonstrated that circEGFR as an oncogene can promote CRC cell viability, invasion, and proliferation.

Previous studies have elucidated that circRNAs as a ceRNAs can sponge miRNAs to alleviate their repression of their target mRNA (32-34). Therefore, we predicted the circEGFR-related miRNAs by using bioinformatics analysis. miR-106a-5p was found to be a target for circEGFR. The miR-106a-5p can inhibit the cell migration and invasion in renal cell carcinoma and osteosarcoma (35,36). Furthermore, it has been reported that miR-106a-5p can inhibit the progression of CRC. However, its role in CRC progression remains unclear. In the present study, we assumed that circEGFR served as a ceRNA by sponging miR-106a-5p to promote the CRC progression. We first examined the circEGFR expression in CRC tissues and their corresponding adjacent normal tissues. The results showed that the expression of circEGFR was upregulated in CRC. Through luciferase reporter assays, we confirmed that circEGFR can bind the miR-106a-5p. In addition, we identified a negative correlation between circEGFR and miR-106a-5p. We also found that the functions of DDX5 could be suppressed by miR-106a-5p. Many studies have reported that DDX5 is associated with many types of cancer, such as prostate cancer (14), gastric cancer (37), and colorectal cancer (38), etc. However, the existing studies have not been sufficient to clarify the underlying mechanism. Here, we investigated the correlations between circEGFR and DDX5 and miR-106a-5p via western blot and qRT-PCR. The results indicated that a positive correlation existed between circEGFR and DDX5, and a negative correlation existed between miR-106a-5p and circEGFR/DDX5, in both overexpression and knockdown contexts. Then, we verified that the expression of these three genes can regulate the CRC progression. Ultimately, we describe a new axis initiated by circEGFR, which is newly confirmed to have oncogenic potential in CRC.

In our study, there are some limitations. For example, our experimental design was not perfect, and there are many details not fully explored. Most importantly, results were all validated at the cellular level, but no *in vivo* experiments were conducted to further deepen the validation. The results obtained so far do not elucidate these molecular mechanisms well; however, they still have some significance to advance to the next step of research.

### Conclusion

Our study identified the relationship between circEGFR and prognosis in CRC and suggested that a new mechanism of circEGFR may serve as an oncogenic factor through the circEGFR/miR-106a-5p/DDX5/AKT axis in CRC.

## Supplementary Material

Click here to view [pdf].

## References

[1] Siegel RL, Miller KD, Fuchs HE, Jemal A (2021). Cancer Statistics, 2021. CA Cancer J Clin.

[2] Siegel RL, Miller KD, Goding Sauer A, Fedewa SA, Butterly LF, Anderson JC (2020). Colorectal cancer statistics, 2020. CA Cancer J Clin.

[3] Siegel RL, Miller KD, Fedewa SA, Ahnen DJ, Meester RGS, Barzi A (2017). Colorectal cancer statistics, 2017. CA Cancer J Clin.

[4] Zhang PF, Wei CY, Huang XY, Peng R, Yang X, Lu JC (2019). Circular RNA circTRIM33-12 acts as the sponge of MicroRNA-191 to suppress hepatocellular carcinoma progression. Mol Cancer.

[B05] Zhang PF, Pei X, Li KS, Jin LN, Wang F, Wu J (2019). Circular RNA circFGFR1 promotes progression and anti-PD-1 resistance by sponging miR-381-3p in non-small cell lung cancer cells. Mol Cancer.

[6] Yu T, Wang Y, Fan Y, Fang N, Wang T, Xu T (2019). CircRNAs in cancer metabolism: a review. J Hematol Oncol.

[7] Memczak S, Jens M, Elefsinioti A, Torti F, Krueger J, Rybak A (2013). Circular RNAs are a large class of animal RNAs with regulatory potency. Nature.

[8] Hansen TB, Jensen TI, Clausen BH, Bramsen JB, Finsen B, Damgaard CK (2013). Natural RNA circles function as efficient microRNA sponges. Nature.

[9] Han K, Wang FW, Cao CH, Ling H, Chen JW, Chen RX (2020). CircLONP2 enhances colorectal carcinoma invasion and metastasis through modulating the maturation and exosomal dissemination of microRNA-17. Mol Cancer.

[10] Pei FL, Cao MZ, Li YF (2020). Circ_0000218 plays a carcinogenic role in colorectal cancer progression by regulating miR-139-3p/RAB1A axis. J Biochem.

[11] Heinlein UA (1998). Dead box for the living. J Pathol.

[12] Jacob J, Favicchio R, Karimian N, Mehrabi M, Harding V, Castellano L (2016). LMTK3 escapes tumour suppressor miRNAs via sequestration of DDX5. Cancer Lett.

[13] Shin S, Rossow KL, Grande JP, Janknecht R (2007). Involvement of RNA helicases p68 and p72 in colon cancer. Cancer Res.

[14] You Z, Liu C, Wang C, Ling Z, Wang Y, Wang Y (2019). LncRNA CCAT1 promotes prostate cancer cell proliferation by interacting with DDX5 and MIR-28-5P. Mol Cancer Ther.

[15] Xu CM, Chen LX, Gao F, Zhu MF, Dai Y, Xu Y (2019). MiR-431 suppresses proliferation and metastasis of lung cancer via down-regulating DDX5. Eur Rev Med Pharmacol Sci.

[16] Jia W, Xu B, Wu J (2018). Circular RNA expression profiles of mouse ovaries during postnatal development and the function of circular RNA epidermal growth factor receptor in granulosa cells. Metabolism.

[17] Qiu BQ, Zhang PF, Xiong D, Xu JJ, Long X, Zhu SQ (2019). CircRNA fibroblast growth factor receptor 3 promotes tumor progression in non-small cell lung cancer by regulating Galectin-1-AKT/ERK1/2 signaling. J Cell Physiol.

[18] Shang BQ, Li ML, Quan HY, Hou PF, Li ZW, Chu SF (2019). Functional roles of circular RNAs during epithelial-to-mesenchymal transition. Mol Cancer.

[19] Xiao H, Liu M (2020). Circular RNA hsa_circ_0053277 promotes the development of colorectal cancer by upregulating matrix metallopeptidase 14 via miR-2467-3p sequestration. J Cell Physiol.

[20] Liu C, Wang L, Jiang Q, Zhang J, Zhu L, Lin L (2019). Hepatoma-derived growth factor and DDX5 promote carcinogenesis and progression of endometrial cancer by activating beta-catenin. Front Oncol.

[21] Nyamao RM, Wu J, Yu L, Xiao X, Zhang FM (2019). Roles of DDX5 in the tumorigenesis, proliferation, differentiation, metastasis and pathway regulation of human malignancies. Biochim Biophys Acta Rev Cancer.

[22] Xue Y, Jia X, Li L, Dong X, Ling J, Yuan J (2018). DDX5 promotes hepatocellular carcinoma tumorigenesis via Akt signaling pathway. Biochem Biophys Res Commun.

[23] Sarkar M, Khare V, Guturi KK, Das N, Ghosh MK (2015). The DEAD box protein p68: a crucial regulator of AKT/FOXO3a signaling axis in oncogenesis. Oncogene.

[24] Wu N, Han Y, Liu H, Jiang M, Chu Y, Cao J (2018). miR-5590-3p inhibited tumor growth in gastric cancer by targeting DDX5/AKT/m-TOR pathway. Biochem Biophys Res Commun.

[25] Mork ME, You YN, Ying J, Bannon SA, Lynch PM, Rodriguez-Bigas MA (2015). High prevalence of hereditary cancer syndromes in adolescents and young adults with colorectal cancer. J Clin Oncol.

[26] Chen L, Zhang S, Wu J, Cui J, Zhong L, Zeng L (2017). circRNA_100290 plays a role in oral cancer by functioning as a sponge of the miR-29 family. Oncogene.

[27] Li Y, Zheng F, Xiao X, Xie F, Tao D, Huang C (2017). CircHIPK3 sponges miR-558 to suppress heparanase expression in bladder cancer cells. EMBO Rep.

[28] Hsiao KY, Lin YC, Gupta SK, Chang N, Yen L, Sun HS (2017). Noncoding effects of circular RNA CCDC66 promote colon cancer growth and metastasis. Cancer Res.

[29] Zhong Y, Du Y, Yang X, Mo Y, Fan C, Xiong F (2018). Circular RNAs function as ceRNAs to regulate and control human cancer progression. Mol Cancer.

[30] Glenfield C, McLysaght A (2018). Pseudogenes provide evolutionary evidence for the competitive endogenous RNA hypothesis. Mol Biol Evol.

[31] Zheng SR, Zhang HR, Zhang ZF, Lai SY, Huang LJ, Liu J (2018). Human papillomavirus 16 E7 oncoprotein alters the expression profiles of circular RNAs in Caski cells. J Cancer.

[32] Wang K, Long B, Liu F, Wang JX, Liu CY, Zhao B (2016). A circular RNA protects the heart from pathological hypertrophy and heart failure by targeting miR-223. Eur Heart J.

[B33] Xu H, Guo S, Li W, Yu P (2015). The circular RNA Cdr1as, via miR-7 and its targets, regulates insulin transcription and secretion in islet cells. Sci Rep.

[34] Liu Q, Zhang X, Hu X, Dai L, Fu X, Zhang J (2016). Circular RNA related to the chondrocyte ECM regulates MMP13 expression by functioning as a MiR-136 'sponge' in human cartilage degradation. Sci Rep.

[35] He QY, Wang GC, Zhang H, Tong DK, Ding C, Liu K (2016). miR-106a-5p suppresses the proliferation, migration, and invasion of osteosarcoma cells by targeting HMGA2. DNA Cell Biol.

[36] Pan YJ, Wei LL, Wu XJ, Huo FC, Mou J, Pei DS (2017). MiR-106a-5p inhibits the cell migration and invasion of renal cell carcinoma through targeting PAK5. Cell Death Dis.

[37] Du C, Li DQ, Li N, Chen L, Li SS, Yang Y (2017). DDX5 promotes gastric cancer cell proliferation *in vitro* and *in vivo* through mTOR signaling pathway. Sci Rep.

[38] Zhang M, Weng W, Zhang Q, Wu Y, Ni S, Tan C (2018). The lncRNA NEAT1 activates Wnt/β-catenin signaling and promotes colorectal cancer progression via interacting with DDX5. J Hematol Oncol.

